# A Missed Opportunity? Exploring Changes in Influenza Vaccination Coverage During the COVID‐19 Pandemic: Data From 12 Countries Worldwide

**DOI:** 10.1111/irv.70057

**Published:** 2025-01-08

**Authors:** Marco Del Riccio, Andrea Guida, Bronke Boudewijns, Susanne Heemskerk, Jojanneke van Summeren, Caroline Schneeberger, Foekje Stelma, Koos van der Velden, Aura Timen, Saverio Caini

**Affiliations:** ^1^ Department of Health Sciences University of Florence Florence Italy; ^2^ Department of Primary and Community Care Radboud University Medical Centre Nijmegen The Netherlands; ^3^ Netherlands Institute for Health Services Research (Nivel) Utrecht The Netherlands; ^4^ Medical School of Specialization in Hygiene and Preventive Medicine University of Florence Florence Italy

**Keywords:** COVID‐19 pandemic, influenza, older adults, respiratory viruses, risk groups, vaccination coverage

## Abstract

**Background:**

Vaccination is a key measure in influenza control, yet global coverage rates remain low, although previous research reported an increase in influenza vaccination coverage rates (VCR) after the onset of the COVID‐19 pandemic. This study aims to assess whether these changes were sustained over time by analyzing VCR trends from 2012 to 2023 in the countries included in the FluCov project.

**Methods:**

Data on influenza VCR from 2012 to 2023 for different age and risk groups were extracted from national health organizations and international sources for countries included in the FluCov project. For coverage rates in the older adults, segmented regression models were used to test if 2020 marked a significant change in VCR trends. Moreover, polynomial regression models were fitted for each country with VCR in the period 2012 to 2020 to predict coverage rates for 2021 to 2023 and to compare these to the actual coverage rates for 2021 to 2023.

**Results:**

For the elderly, we retrieved influenza VCR data for 12 countries. In 2020, VCR among elderly increased in 10 countries, but the increase was statistically significant in Spain and England only. Moreover, all countries except Spain reverted to levels within the confidence intervals of trends modeled using pre‐2020 data.

**Conclusions:**

Although influenza VCR increased in 2020, these changes were statistically significant in only two out of 12 countries, and no consistent, sustained increase was observed afterward, except in Spain. The findings suggest the need for continuous monitoring of VCR and the implementation of strategies to promote and maintain high vaccination coverage rates.

## 1 Introduction

Seasonal influenza leads every year to significant morbidity and is estimated to be responsible for a minimum of 500,000 deaths worldwide [1]; some populations, such as older adults, children, pregnant women, and individuals with cardiometabolic risk factors, may be disproportionately affected and more often suffer from serious respiratory, cardiovascular, and inflammatory complications as result of an influenza infection [2].

Influenza vaccination has been shown to reduce both influenza infection rates and related complications especially in such high‐risk groups [3, 4]; it is therefore one of the key components of the strategic objectives of the global influenza strategy (2019–2023) [5] and it is recommended to be repeated annually. The World Health Organization (WHO) strategic advisory group of experts (SAGE) on immunization has recommended seasonal influenza vaccination as a high priority for five high‐risk target groups (health workers, pregnant women, children, elderly, and people living with chronic conditions) [6]; the European Council recommends a target seasonal influenza vaccination rate of 75% in the elderly (65 years and above) and in persons aged > 6 months with a chronic medical condition [7]. However, global coverage rates remain below recommended levels, likely influenced by different factors including gaps in vaccine literacy, pre‐existing beliefs regarding vaccination, safety concerns, and other inertial factors (such as organizational barriers) ultimately contributing to vaccine hesitancy [8, 9, 10].

In the season immediately following the onset of the COVID‐19 pandemic (2020 in southern hemisphere, 2020/2021 in northern hemisphere), a noticeable increase in influenza vaccination coverage rates (VCR) was observed across several countries [11]. This rise can be attributed to different  reasons: on one hand, people might have become more aware of the risks associated with respiratory infections in the early phases of the pandemic, which likely led to an increased sense of urgency in getting vaccinated against influenza [12]. Moreover, understanding the potential complications of being infected by either influenza or COVID‐19 (or worse, simultaneously by both) likely pushed individuals to prioritize influenza prevention measures, especially considering that no vaccine against COVID‐19 was available until the end of the season. In addition, governments and health organizations invested considerable resources into promoting influenza vaccination, thereby emphasizing the importance of preventing respiratory infections in order to reduce the burden of respiratory infections on healthcare systems [13]. However, despite previous studies showing an initial global increase in influenza VCR since the start of the COVID‐19 pandemic in 2020, there is a lack of research exploring how trends changed in subsequent seasons. In 2021, FluCov, a global initiative aimed at understanding and communicating the impact of COVID‐19 on influenza activity and influenza VCR, was launched and encompassed data from 22 countries [14]. The aim of the present study, embedded in the FluCov project, is to describe influenza VCR in 22 countries before (2012–2019) and during (2020–2023) the COVID‐19 pandemic in different age and risk groups. In particular, our aim was to understand whether a significant, sustained increase in influenza VCR persisted after 2020 or if coverage rates reverted to pre‐COVID‐19 levels, by answering two specific questions:

- Was 2020 a significant breakpoint year, indicating a significant change in VCR in the years after, either through a sustained increase or by stabilizing at levels significantly higher than pre‐COVID?
- Are current VCR significantly different from the rates predicted using models based on pre‐2020 data?

## 2 Methods

### 2.1 Data Collection

This descriptive study is part of the FluCov project [14]. At the time of data collection, the FluCov project encompassed 22 countries worldwide, including Brazil, Canada, Mexico, the United States, England, France, Germany, Israel, Italy, Netherlands, Poland, Spain, India, Philippines, Thailand, Vietnam, Australia, China, Japan, South Korea, South Africa, and Egypt. Data on influenza VCR from 2012 onwards was collected until October 2024 from national health organizations, such as national public health agencies and governmental websites. Whenever these data were unavailable, we searched websites and reports from international organizations such as WHO‐UNICEF, the Organization for Economic Co‐operation and Development (OECD), the European Centre for Disease Prevention and Control (ECDC), and OurWorldInData (File S1). VCR for different age and risk groups were collected, along with specific definitions for how each group was defined by each country. The groups included older adults, pregnant women, healthcare professionals, children, risk groups with indications for vaccination, and others (e.g., “adults,” if monitored in a given country). In some cases, such as in the Netherlands, coverage data for older adults were available for both the 60+ and 65+ groups. To ensure comparability with other countries, we consistently used the 65+ definition when available, as it is the most common standard. Data were collected for each risk group the way it is monitored in a country, disregarding whether the vaccination is offered for free or not. To reflect the timing differences in influenza outbreaks and the organization of vaccination campaigns across different regions, from now on when we refer to a specific year (e.g., 2022) for VCR, we imply the calendar year for southern hemisphere and intertropical belt countries, and the first year of the influenza season for northern hemisphere countries, where influenza season spans two calendar years (e.g., 2022/2023).

### 2.2 Statistical Analysis

VCR were collected and organized in a dedicated spreadsheet by three researchers (MDR, AG, BB) who also double‐checked each other's entries. Data were then presented in tables for different age and risk groups. For older adults, the group for which we found the most data across the largest number of countries, we also aimed to answer two key questions consistent with literature indicating an increase in VCR in 2020. We specifically aimed to determine if 2020 marked a significant shift in VCR (breakpoint year) and whether any observed increase was simply an outlier, showing temporary deviations from pre‐2020 trends that ultimately reverted to the original patterns. This same analysis would not have been possible for other groups because of insufficient data. To answer the first question, we used segmented regression models to test the hypothesis that 2020 marked a significant change in VCR. In particular, we used the “segmented” function to model piecewise‐linear relationships and identify whether a given country saw a change in the slope of coverage rates in 2020. Then, we used the “stepmented” function to assess whether coverage rates experienced an abrupt change in 2020, suggesting an immediate and noticeable impact rather than a gradual trend shift [15].

For the second question, we employed polynomial regression to model the trend in VCR for years following 2021 (2021–2023), using as input the data up to 2020 and excluding the data from 2020 onwards. We then compared this modeled trend to the actual trend by producing charts that visually represented the differences in influenza VCR over time (actual vs. modeled trend). If the actual coverages stayed within the confidence intervals of the predicted modeled coverage, it indicated that no significant change with regard to the pre‐COVID situation was found. Conversely, if the actual trend and the modeled trend differed significantly, with actual values falling outside the predicted confidence intervals, it suggested that there was a significant change in VCR trends post‐2020. To determine the optimal degree of the polynomial regression model for each country, we fitted polynomial models of degrees 1, 2, and 3 to the pre‐COVID‐19 pandemic data (thus, excluding years 2020 and later) and selected the model with the lowest Akaike information criterion (AIC) value, indicating the best trade‐off between model complexity and goodness of fit [16]. The goodness of fit for both segmented and stepmented regression models and for the polynomial regression model was evaluated using *R*‐squared values, which indicate the proportion of variance explained by the models. Australia and Japan were excluded from this further analysis because of the paucity of coverage rate data before 2020 (Australia) and after 2020 (Japan), which prevented reliable modeling and comparison between actual and modeled rates.

### 2.3 Software

All analyses were conducted using Rstudio version 2023.06.0+421 (Posit Software, PBC, Boston, MA, USA, http://www.posit.co/.) and in particular the package “segmented” [15].

## 3 Results

### 3.1 Older Adults

**FIGURE 1:**
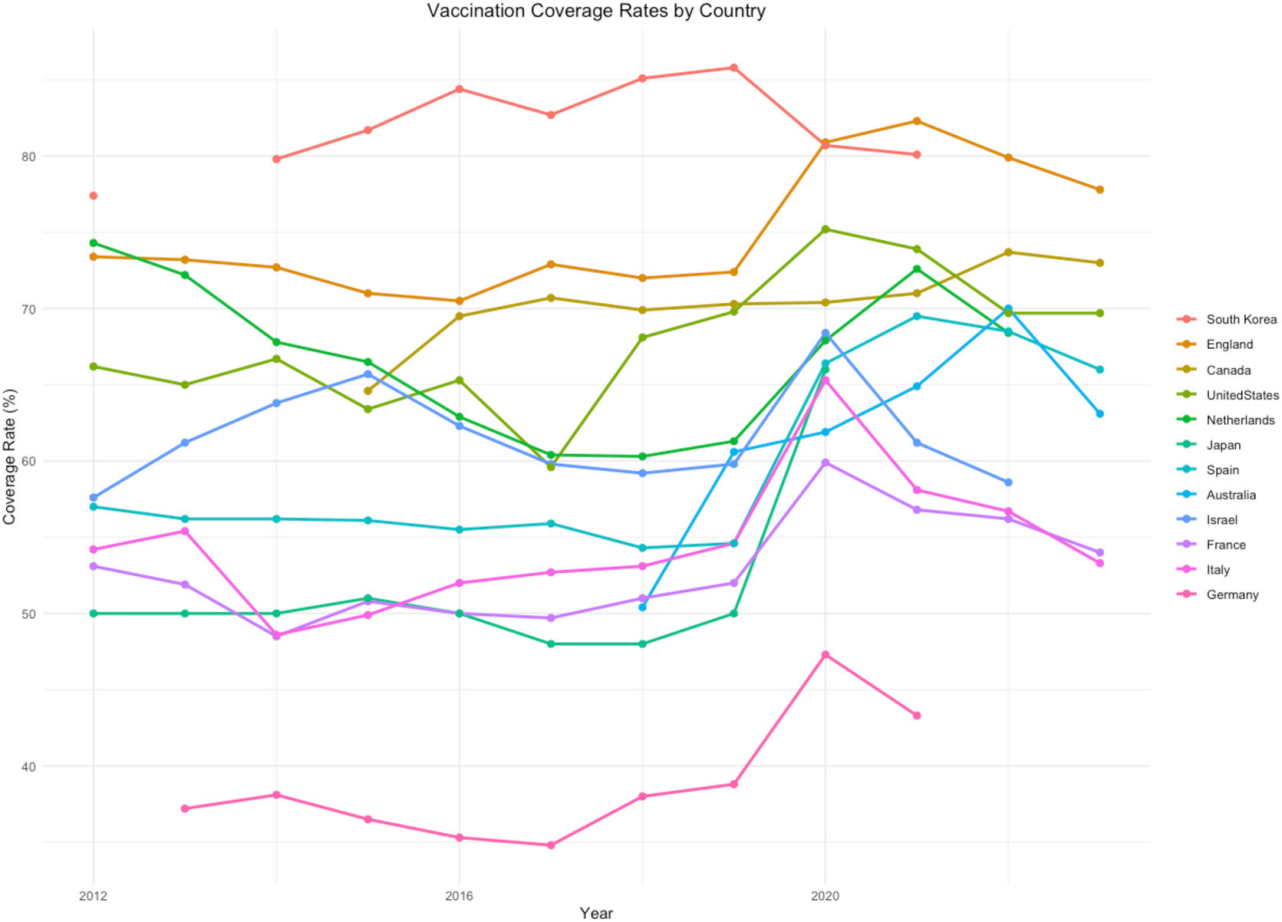
Influenza vaccination coverage rates (%) for Older Adults (60 + or 65+) from 2012 to 2023.

Out of 22 countries, we retrieved influenza VCR data for older adults in 12 (Australia, Canada, England, France, Germany, Israel, Italy, Japan, Netherlands, South Korea, Spain, the United States). For the remaining 10 countries, either data were unavailable, or the information lacked clarity on the calculation of numerators and denominators (Figure 1, Table 1). Sources from which we draw the data are reported in the supporting information (File S1), together with latest available influenza recommendations in the 12 countries (File S2).

**TABLE 1:** Influenza vaccination coverage rates (%) for older adults (60+ or 65+) from 2012 to 2023.

Country	Age	Vaccination coverage rate per season											
		2012	2013	2014	2015	2016	2017	2018	2019	2020	2021	2022	2023
Australia	65+	—	—	—	—	—	—	50.4	60.6	61.9	64.9	70.0	63.1
Canada	65+	—	—	—	64.6	69.5	70.7	69.9	70.3	70.4	71	73.7	73.0
England	65+	73.4	73.2	72.7	71	70.5	72.9	72	72.4	80.9	82.3	79.9	77.8
France	65+	53.1	51.9	48.5	50.8	50	49.7	51	52	59.9	56.8	56.2	54.0
Germany	60+	—	37.2	38.1	36.5	35.3	34.8	38	38.8	47.3	43.3	—	—
Israel	65+	57.6	61.2	63.8	65.7	62.3	59.8	59.2	59.8	68.4	61.2	58.6	—
Italy	65+	54.2	55.4	48.6	49.9	52	52.7	53.1	54.6	65.3	58.1	56.7	53.3
Japan	65+	50	50	50	51	50	48	48	50	66	—	—	—
Netherlands	65+	74.3	72.2	67.8	66.5	62.9	60.4	60.3	61.3	67.9	72.6	68.4	—
South Korea	65+	77.4	—	79.8	81.7	84.4	82.7	85.1	85.8	80.7	80.1	—	—
Spain	65+	57	56.2	56.2	56.1	55.5	55.9	54.3	54.6	66.4	69.5	68.5	66.0
United States	65+	66.2	65	66.7	63.4	65.3	59.6	68.1	69.8	75.2	73.9	69.7	69.7

Influenza VCRs in older adults across various countries from 2012 to 2023 exhibit diverse trends. Many countries, including England, France, Germany, Israel, Italy, Japan, Netherlands, Spain, and the United States, saw an increase in VCR in 2020; however, most of them experienced a decline already starting in 2021 (France, Germany, Israel, Italy, and the United States), and in general in the first season after the peak of COVID‐19 cases was passed. Australia, as an example, had most COVID‐19 hospitalizations during the first half of 2022 (peak of hospitalizations in July 2022), and this period corresponded with influenza vaccination campaign in 2022 and the highest reached VCR [17]. Notably, Canada, Netherlands, and Spain have maintained relatively high vaccination rates even after the initial increase.

The segmented regression analysis across multiple countries shows that 2020 is not statistically significant as a breakpoint for changes in vaccination coverage among the elderly. In particular, none of the country‐specific models showed statistical significance in their coefficients to support 2020 as a critical year of change in the VCR slope (coefficients not shown). The stepmented analysis identified significant estimated breakpoints in VCRs between 2019 and 2020 for England and Spain. In England, a significant breakpoint was found in 2019 (*p* < 0.001). Similarly, Spain showed a significant breakpoint in 2019 (*p* < 0.001). This means that both countries experienced abrupt and significant shifts in VCRs around 2019, indicating a notable change in their vaccination trends between 2019 and 2020, and identifying 2020 as a breakpoint year. Overall, the hypothesis that 2020 represents a significant breakpoint in elderly vaccination coverage is only supported for Spain and England. Based on R‐squared values, both models had moderate to high goodness of fit for all countries except Israel.

**FIGURE 2:**
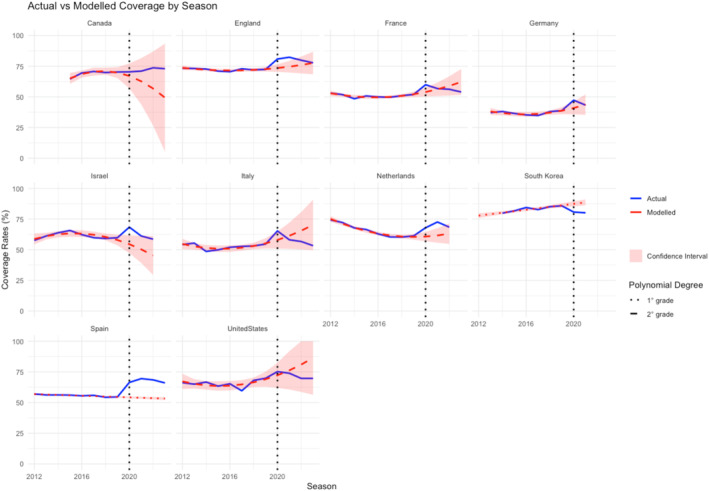
Actual versus modeled influenza vaccination coverage rates (VCR) for Older Adults (2012–2023). Australia and Japan were excluded from this model due to the paucity of data. The blue lines represent the actual VCR, while the red dashed lines represent the modeled VCR based on pre‐2020 data. The red line is noncontinuous: It is dashed when a second‐degree polynomial is used for regression, and dotted when a first‐degree polynomial is used. The light red areas represent confidence intervals.

Figure 2 shows the results of the actual versus predicted influenza VCR for older adults across the analyzed countries from 2012 to 2022 (or 2023 for those countries where recent data was available).

Comparing actual versus modeled trends, it is possible to note how within 2 years after 2020, almost all countries reverted to levels within the confidence intervals of the pre‐existing modeled trends. Spain is the only significant exception, with a predicted VCR of 53.6% (95%CI 52.5–54.6) versus actual VCR of 68.5%. In terms of model goodness of fit, only United States showed low R‐squared (< 0.50), whereas all other countries exhibited moderate to very high fit. Notably, in Spain and South Korea, the VCR trends before 2020 followed a nearly linear pattern: Therefore, based on the AIC, a linear regression model was applied, resulting in tight confidence intervals due to the data consistency and reduced uncertainty around the trend line.

### 3.2 Other Groups

The detailed VCRs for healthcare workers, pregnant women, children, populations with comorbidities, and adults across selected countries from 2012 to 2023 are reported in Table 2.

**TABLE 2:** Influenza vaccination coverage rates (VCR) for various groups across selected countries (2012–2023).

Healthcare workers													
	Group details	2012	2013	2014	2015	2016	2017	2018	2019	2020	2021	2022	2023
England		45.6	54.8	58.3	53.9	67.3	68.7	70.3	74.3	76.8	61.4	49.4	
France											28.0	25.0	22.0
Germany											51.0	59.0	58.0
Spain							31.1	35.0	39.1	65.6	60.0	50.8	44.0
United States		72.0	75.2	77.3	79.0	78.6	78.4	81.1	80.7	75.9	79.9	75.9	75.4

Pregnant women													
	Group details	2012	2013	2014	2015	2016	2017	2018	2019	2020	2021	2022	2023
Canada								45.0		45.0	45.0	53.0	
England		40.3	39.8	44.1	42.3	44.9	47.2	45.2	43.7	43.6	37.9	35.0	
Germany						10.0			16.6	23.0	18.0		
Israel								32.0		35.1	26.2		
South Korea					44.8	31.9	44.1	52.0	68.5				
Spain						27.2	29.6	40.6	50.0	62.3	55.3	56.0	58.0
United States		50.5	52.5	50.3	49.9	53.6	49.1	53.7	61.2	54.5	49.6	47.2	

Children													
	Group details	2012	2013	2014	2015	2016	2017	2018	2019	2020	2021	2022	2023
Australia	6m‐5y									46.1	26.5	34.1	28.3
Australia	5y‐14y									27.4	14.5	23.2	16.1
England	2y‐3y			39.9	36.6	40.2	44	44.9	43.8	56.7	50.1	43.7	44.4
England	4y‐11y								60.4	62.5	57.4	56.3	
Italy	6m‐23m	1.5	1.3	1.1	1.1	1.5	1.4		2.8	9.2	7.0	7.2	9.8
Italy	2y‐4y	2.6	2.5	1.8	1.8	2.6	2.4		4.2	19.0	17.4	9.2	16.7
Italy	5y‐8y	2.6	2.6	1.9	1.8	2.4	2.2		3.1	13.1	12.2	22.6	11.7
Italy	9y‐14y	2.0	2.1	1.5	1.4	1.8	1.8		1.9	6.0	4.4	4.9	4.6
Italy	15y‐17y	2.1	2.3	1.5	1.6	1.9	2.5		1.9	4.5	2.5	2.1	1.9
South Korea	0y‐12y				62.5	61.4	66.2	71.3	83.1				
United States	6m4y	69.8	70.4	70.4	70.0	70.0	67.8	73.4	75.5	68.0	66.7	65.6	64.2
United States	5y‐17y				55.9	55.6	54.8	59.1	60.2	55.8	55.1	55.1	
United States	6m17y	56.6	58.9	59.3	59.3	59	57.9	62.6	63.8	58.6	57.8	57.4	55.4

Population with medical conditions with indication for vaccination													
	Group details	2012	2013	2014	2015	2016	2017	2018	2019	2020	2021	2022	2023
Canada	18–64				37.2	37	39.4	42.8	43.6	40.5	37.6	43.1	44.0
Germany	18+								32.2	39			
Netherlands	< 60				35.3	32.7	29.4	30.8	32	25	27	30.1	27.9
England	6m‐64y	51.3	52.3	50.3	45.1	48.6	49.7	48	44.9	53	52.9	49.1	41.4
France	< 65				39.1	28.7	28.9	29.2	31	38.7	34.3	31.6	27.5
South Korea	13y‐64y				32.3	38.1	35.2	34.8	41.9				
United States	18–64	47.0	46.3	47.6	46.0	46.4	38.8	47.9	51.4	51.0	50.4	48.8	45.5

All adults, disregarding their health conditions													
	Group details	2012	2013	2014	2015	2016	2017	2018	2019	2020	2021	2022	2023
Australia	50–64									35.2	38.2	46.8	36.8
Canada	18+				34.3	35.8	38.3	41.8	41.8	40.4	38.7	43.0	42.0
Italy	18–44	2.1	2.5	1.9	1.8	2.2	2.2		3.1	5.9	4.2	4.1	3.7
Italy	45–64	9.0	9.5	7.5	7.7	8.5	8.7		9.6	16.8	13.7	13.3	11.8
Netherlands	18+						21.4	22.1	22.4	25.5			
Netherlands	18–64						10.0	10.4	10.7	12.1			
United States	18+	41.5	42.2	43.6	41.7	43.3	37.1	45.3	48.4	50.2	49.4	46.9	44.9
United States	18–64	35.7	36.7	38.0	36.3	37.5	31.1	39.0	42.3	43.0	42.0	40.0	37.0

Among healthcare workers, England's VCR gradually increased from 53.9% in 2015 to 76.8% in 2020 and then started declining (49.4% in 2023). Spain peaked at 65.6% in 2020, maintaining around 50.8% in 2022. The United States consistently showed high rates, peaking at 81.1% in 2018 and remaining above the 75% threshold since 2013. For pregnant women, England saw a drop after 2020, as well as Spain, that increased consistently from 27.2% in 2015 to 62.3% in 2020 and then declined to 53.6% in 2022. The United States reached its peak in 2019 (61.2%), after which VCR started declining (47.2% in 2022).

In England, VCR in children aged 2 to 3 years increased from 36.6% in 2015 to 56.7% in 2020, then declined to 43.7% in 2022. The same happened in Italy, with different age groups reaching the maximum coverage rates in 2020, before starting to decline. The US saw high coverage for children aged 6 months to 7 years, peaking at 63.8% in 2019 and slightly declining to 57.4% in 2023. For adults and populations with comorbidities, mixed patterns are visible across different countries; in particular, France, England, and the United States reported decreasing coverage rates after 2020/21.

## 4 Discussion

Our study aimed to collect data and assess trends in influenza VCR in older adults and other risk groups in 22 countries—part of the FluCov project [14]—from 2012 to 2023. In particular, considering early pandemic data suggested increased willingness for influenza vaccination, we aimed to determine if 2020 marked a significant breakpoint year for VCR [11, 18, 19]. Our results show that although some countries experienced increases in VCR throughout different age and risk groups around 2020, these changes were not significant, nor consistently sustained in subsequent years. Our analysis indicates that the onset of the COVID‐19 pandemic did not serve as a breakpoint moment, but rather as an outlier year: After 2020, VCR progressively reverted to their usual trends and decreased for most countries and in most age and risk groups. For example, France, Germany, Israel, Italy, and the United States saw increases in 2020 in VCR in older adults, followed by declines in the following years. In England, the stepmented regression analysis yielded significant results, indicating an abrupt change in VCRs between 2019 and 2020. However, when predicting VCR for 2021 to 2023 based on pre‐2020 data and comparing these predicted rates with actual coverage rates for 2021 to 2023, we cannot conclude that this increase is significantly sustained over the long term, as the actual coverage rates eventually fell within the confidence intervals of the predicted trends. This pattern was also observed across other groups, including healthcare workers, pregnant women, and children, with the uptrend that seems to have been maintained only by few countries. Notably, in Spain, the VCR for older adults showed a significant and sustained increase following 2020. Unlike other countries where the VCR reverted to levels within the confidence intervals of pre‐existing trends, Spain maintained higher coverage rates, indicating a potentially more robust and lasting response to vaccination efforts during this period. However, this conclusion remains tentative, as reports from 2023 indicate a slight decrease in VCR among older adults in Spain, reaching the lowest rate in the past 4 years [20], making it important to confirm these results in the coming years to understand whether VCRs in Spain are gradually declining or stabilizing at a new level.

Across many parts of the world, regions effectively adjusted their vaccine delivery methods to sustain influenza vaccination services at the beginning of the COVID‐19 pandemic, differently from what happened, as an example, with other routine childhood vaccines [21]: by implementing innovative strategies such as modified vaccination sites and flexible scheduling, they ensured continued access to and uptake of vaccines [13, 22, 23]. However, and despite an initial increase in influenza VCR in the early phases of the pandemic, other studies have shown that these initial increases in influenza VCR were not sustained in the following years, consistently with our results. For example, Ma and colleagues [24] highlighted how influenza vaccination coverage among healthcare workers in China declined after 2020, as resources were redirected toward COVID‐19 vaccination efforts and accessibility to influenza vaccines decreased. Similarly, Cunniff and colleagues reported significant declines in influenza vaccination rates in the United States during the pandemic, as participants showed decreased intention to receive routine vaccines, such as the influenza vaccine [25]. Finally, a RAISE group study found a temporary increase in VCR among older adults in some European countries and Israel during the first pandemic year, followed by declines in most by 2021/2022, with lower rates persisting in Central and Eastern Europe despite efforts to improve access [26].

Identifying the precise reasons for the decline in influenza VCRs following their initial increase during the pandemic is beyond the scope of this ecological study. However, the recent literature reports several factors that may have contributed to this trend. One potential reason is pandemic fatigue, and in particular vaccine fatigue, where the intense focus on COVID‐19 vaccination efforts may have led to a general sense of exhaustion and reluctance toward receiving additional vaccines, including influenza vaccines [27]. Moreover, after an initial increase in fear toward respiratory infections and a heightened risk perception due to overwhelmed health systems, the situation might gradually have returned to normal. As a consequence, a reduced risk perception regarding influenza may have resulted in complacency, as the additional individuals who decided to get vaccinated after the onset of the COVID‐19 pandemic may no longer perceive seasonal influenza as a significant enough threat to warrant vaccination [28]. Additionally, vaccine hesitancy and misinformation may have further contributed, as the spread of false information about vaccine safety and efficacy during the pandemic heightened general skepticism toward vaccines. This misinformation often creates doubts and fears, deterring individuals from getting vaccinated not only for COVID‐19 but also for influenza [29, 30]. If the pandemic was initially highlighted as a window of opportunity to promote influenza vaccination, this now seems like a missed opportunity. This momentum could have served as a boost to reinforce influenza vaccination and underline the importance of vaccines. However, this momentum appears not to have been fully realized to its potential.

The study has a number of limitations: First, data for older adults were available for only 12 countries, and even fewer countries provided data for other age and risk groups, which may affect the generalizability of the findings. Moreover, many factors should be considered when interpreting vaccination coverage data, which was not feasible in our ecological study. The low‐moderate goodness of fit observed in the segmented regression models may reflect the influence of unaccounted factors, such as changes in national vaccination policies, public health campaigns, and societal attitudes toward vaccination over time; this highlights the need for cautious interpretation of VCR variations and also reinforces the importance of this study in examining these trends. Furthermore, due to differences in data collection methods and the heterogeneity of data sources across countries, comparisons between countries should be made cautiously, as variations in reporting practices may affect consistency; comparisons within the same country over different years, therefore, should be preferred. The study would benefit from more post‐COVID‐19 years of data, enhancing the reliability and validity of our results and providing a more accurate understanding of vaccination coverage trends. Finally, due to statistical uncertainty—particularly in countries with fluctuating historical coverage like the United States—confidence intervals may suggest trend reversion but also reflect statistical uncertainty. Thus, interpretations should cautiously balance these uncertainties. However, to our knowledge, this is the first study that analyzed influenza VCR in different age and risk groups in 22 countries over more than a decade (2012–2023), with a specific focus on the impact of the COVID‐19 pandemic. Among the strengths of this study is its extensive dataset, which covers multiple age groups and risk factors, while primarily focusing on the over‐65 population for quantitative analysis, as this group provided the most consistent data across countries. Additionally, we employed refined statistical methods to test our hypotheses, providing a robust framework for understanding the data beyond presentation in tables.

In conclusion, our study provides a comprehensive analysis of influenza VCR across different age and risk groups in 12 countries during the COVID‐19 pandemic, highlighting how no sustained changes seem to have occurred in VCRs, despite a sensible increase in the first phase of the pandemic. This represents a missed opportunity to capitalize on the initial increase in vaccination uptake, underscoring the need for more robust efforts to promote influenza vaccination, especially in high‐risk groups. Furthermore, we advocate for a coordinated global effort to improve the monitoring of vaccination programs and ensure transparency in data reporting: This would facilitate more accurate tracking of coverage rates, allow for timely interventions, and improve public health outcomes by increasing VCRs. In facts, addressing current inconsistencies in data monitoring and reporting shall represent a key objective in the future, as clear, standardized, and comprehensive data collection methods are essential for reliable assessments and comparisons across countries.

## Author Contributions

**Marco Del Riccio:** conceptualization, data curation, formal analysis, methodology, visualization, writing – original draft, writing – review and editing. **Andrea Guida:** data curation, writing – review and editing. **Bronke Boudewijns:** data curation, writing – review and editing**. Susanne Heemskerk:** data curation, writing – review and editing**. Jojanneke van Summeren:** methodology, supervision, writing – review and editing**. Caroline Schneeberger:** project administration, writing – review and editing**. Foekje Stelma:** funding acquisition, project administration, writing – review and editing. **Koos van der Velden:** conceptualization, supervision, writing – review and editing. **Aura Timen:** conceptualization, supervision, writing – review and editing. **Saverio Caini:** conceptualization, methodology, supervision, writing – review and editing.

## Conflicts of Interest

The authors declare no conflicts of interest.

## Supporting information


**File S1.** Sources of Influenza vaccination coverages.

**File S2.** Influenza vaccine recommendations for season 2023/24 (or most recent season, if 2023/24 was not available).

## Data Availability

All data used in this study were retrieved from publicly available sources, including national health organizations and international databases. Detailed sources for each country's influenza vaccination coverage rates are provided within the supporting information.
