# Supplementary material for: A Missed Opportunity? Exploring Changes in Influenza Vaccination Coverage During the COVID‐19 Pandemic: Data From 12 Countries Worldwide
Source: Influenza Other Respir Viruses. 2025 Jan 8;19(1):e70057. doi: 10.1111/irv.70057 (PMC11710886; doi:10.1111/irv.70057)
Supplement: Supplementary file 1 — File S1. Sources of Influenza vaccination coverages. [file IRV-19-e70057-s001.docx]

**Supplementary file 1: Sources of Influenza vaccination coverages**

**Australia**

- Older adults
  - Department of Health, Australia
  - <https://ncirs.org.au/influenza-vaccination-coverage-data/historical-national-influenza-vaccination-coverage-2020-2022>
- Children
  - <https://ncirs.org.au/influenza-vaccination-coverage-data/historical-national-influenza-vaccination-coverage-end-year-age>
- Adults
  - <https://ncirs.org.au/influenza-vaccination-coverage-data>

**Canada**

- Older adults
  - <https://www.canada.ca/en/public-health/services/immunization-vaccines/vaccination-coverage.html>
- Pregnant women
  - <https://www.canada.ca/en/services/health/publications/vaccines-immunization.html>
- Population with medical conditions with indication for vaccination
  - <https://www.canada.ca/en/public-health/services/immunization-vaccines/vaccination-coverage.html>
- Adults
  - <https://www.canada.ca/en/public-health/services/immunization-vaccines/vaccination-coverage.html>

**England**

- Older adults
  - <https://fingertips.phe.org.uk/search/flu%20vaccination#page/4/gid/1/pat/159/par/K02000001/ati/15/are/E92000001/iid/30314/age/27/sex/4/cat/-1/ctp/-1/yrr/1/cid/4/tbm/1>
- Healthcare workers
  - <https://fingertips.phe.org.uk/search/flu%20vaccination#page/4/gid/1938132917/pat/159/par/K02000001/ati/15/are/E92000001/iid/92461/age/-1/sex/-1/cat/-1/ctp/-1/yrr/1/cid/4/tbm/1>
- Pregnant women
  - <https://www.gov.uk/government/collections/vaccine-uptake>
- Children
  - <https://fingertips.phe.org.uk/search/flu%20vaccination#page/4/gid/1000043/pat/159/par/K02000001/ati/15/are/E92000001/iid/93386/age/243/sex/4/cat/-1/ctp/-1/yrr/1/cid/4/tbm/1>
  - <https://fingertips.phe.org.uk/search/flu%20vaccination#page/4/gid/1000043/pat/159/par/K02000001/ati/15/are/E92000001/iid/93691/age/176/sex/4/cat/-1/ctp/-1/yrr/1/cid/4/tbm/1>
- Population with medical conditions with indication for vaccination
  - <https://fingertips.phe.org.uk/search/flu%20vaccination#page/4/gid/1938133210/pat/159/par/K02000001/ati/15/are/E92000001/iid/30315/age/226/sex/4/cat/-1/ctp/-1/yrr/1/cid/4/tbm/1>

**France**

- Older adults
  - <https://www.santepubliquefrance.fr/determinants-de-sante/vaccination/articles/donnees-regionales-de-couverture-vaccinale-grippe-par-saison-et-dans-chaque-groupe-d-age>
- Population with medical conditions with indication for vaccination
  - <https://www.santepubliquefrance.fr/determinants-de-sante/vaccination/articles/donnees-regionales-de-couverture-vaccinale-grippe-par-saison-et-dans-chaque-groupe-d-age>

**Germany**

- Older adults
  - <https://data.oecd.org/healthcare/influenza-vaccination-rates.htm>
  - https://www.ecdc.europa.eu/sites/default/files/documents/seasonal-influenza-vaccination-coverage-recommendations.pdf
- Pregnant women
  - <https://www.rki.de/DE/Home/homepage_node.html>
  - https://www.ecdc.europa.eu/sites/default/files/documents/seasonal-influenza-vaccination-coverage-recommendations.pdf
- Population with medical conditions with indication for vaccination
  - <https://www.rki.de/SharedDocs/FAQ/Impfen/Influenza/Hochdosis-Impfstoffe/FAQ_Uebersicht.html?nn=2370434>
  - https://www.ecdc.europa.eu/sites/default/files/documents/seasonal-influenza-vaccination-coverage-recommendations.pdf

**Israel**

- Older adults
  - <https://data.oecd.org/healthcare/influenza-vaccination-rates.htm>
- Pregnant women
  - <https://immunizationdata.who.int/pages/coverage/flu.html?CODE=ISR&ANTIGEN=&YEAR=>
- Children
  - https://immunizationdata.who.int/pages/coverage/flu.html?CODE=ISR&ANTIGEN=&YEAR=

**Italy**

- Older adults
  - <https://www.salute.gov.it/portale/influenza/dettaglioContenutiInfluenza.jsp?lingua=italiano&id=679&area=influenza&menu=vuoto>
- Children
  - <https://www.salute.gov.it/portale/influenza/dettaglioContenutiInfluenza.jsp?lingua=italiano&id=679&area=influenza&menu=vuoto>
- Adults
  - <https://www.salute.gov.it/portale/influenza/dettaglioContenutiInfluenza.jsp?lingua=italiano&id=679&area=influenza&menu=vuoto>

**Japan**

- Older adults
  - <https://www.oecd-ilibrary.org/social-issues-migration-health/influenza-vaccination-rates/indicator/english_e452582e-en>

**Netherlands**

- Older adults
  - <https://www.nivel.nl/nl/publicatie/vaccine-coverage-dutch-national-influenza-prevention-program-2022-brief-monitor>
  - https://www.nivel.nl/en/publicatie/vaccine-coverage-dutch-national-pneumococcal-vaccination-program-adults-2023-brief
- Population with medical conditions with indication for vaccination
  - <https://www.nivel.nl/nl/publicatie/vaccine-coverage-dutch-national-influenza-prevention-program-2022-brief-monitor>
  - https://www.nivel.nl/en/publicatie/vaccine-coverage-dutch-national-pneumococcal-vaccination-program-adults-2023-brief
- Adults
  - <https://www.nivel.nl/nl/publicatie/vaccine-coverage-dutch-national-influenza-prevention-program-2022-brief-monitor>
  - https://www.nivel.nl/en/publicatie/vaccine-coverage-dutch-national-pneumococcal-vaccination-program-adults-2023-brief

**South Korea**

- Older adults
  - <https://www.oecd-ilibrary.org/social-issues-migration-health/influenza-vaccination-rates/indicator/english_e452582e-en>
- Pregnant women
  - <https://journals.plos.org/plosone/article?id=10.1371/journal.pone.0262594>
- Children
  - <https://journals.plos.org/plosone/article?id=10.1371/journal.pone.0262594>
- Population with medical conditions with indication for vaccination
  - <https://journals.plos.org/plosone/article?id=10.1371/journal.pone.0262594>

**Spain**

- Older adults
  - <https://pestadistico.inteligenciadegestion.sanidad.gob.es/publicoSNS/I/sivamin/informe-de-evolucion-de-coberturas-de-vacunacion-por-vacuna>
  - <https://es.statista.com/estadisticas/617835/cobertura-de-vacunacion-antigripal-en-adultos-mayores-por-temporadas-espana/>
  - https://www.ecdc.europa.eu/sites/default/files/documents/seasonal-influenza-vaccination-coverage-recommendations.pdf
- Healthcare workers
  - <https://pestadistico.inteligenciadegestion.sanidad.gob.es/publicoSNS/I/sivamin/informe-de-evolucion-de-coberturas-de-vacunacion-por-vacuna>
  - https://www.ecdc.europa.eu/sites/default/files/documents/seasonal-influenza-vaccination-coverage-recommendations.pdf
- Pregnant women
  - <https://gateway.euro.who.int/en/indicators/infl_9-influenza-vaccination-coverage-pregnant-women/#id=31629>
  - <https://pestadistico.inteligenciadegestion.sanidad.gob.es/publicoSNS/I/sivamin/informe-de-evolucion-de-coberturas-de-vacunacion-por-vacuna>
  - https://www.ecdc.europa.eu/sites/default/files/documents/seasonal-influenza-vaccination-coverage-recommendations.pdf

**United States**

- Older adults
  - <https://www.cdc.gov/flu/fluvaxview/index.htm>
- Healthcare workers
  - <https://www.cdc.gov/flu/fluvaxview/links.htm#health-care>
- Pregnant women
  - <https://www.cdc.gov/flu/fluvaxview/links.htm#pregnant>
- Children
  - <https://www.cdc.gov/flu/fluvaxview/links.htm#general>
- Population with medical conditions with indication for vaccination
  - <https://www.cdc.gov/flu/fluvaxview/dashboard/vaccination-adult-coverage.html>
- Adults
  - <https://www.cdc.gov/flu/fluvaxview/links.htm#general>
