# Supplementary material for: A Missed Opportunity? Exploring Changes in Influenza Vaccination Coverage During the COVID‐19 Pandemic: Data From 12 Countries Worldwide
Source: Influenza Other Respir Viruses. 2025 Jan 8;19(1):e70057. doi: 10.1111/irv.70057 (PMC11710886; doi:10.1111/irv.70057)
Supplement: Supplementary file 2 — File S2. Influenza vaccine recommendations for season 2023/24 (or most recent season, if 2023/24 was not available). [file IRV-19-e70057-s002.docx]

**Supplementary file 2: Influenza vaccine recommendations for season 2023/24 (or most recent season, if 2023/24 was not available)**

**Disclaimer:** The following appendix contains information on influenza vaccination recommendations from various countries. It is important to note that the details provided may differ between countries. In some instances, the information pertains to groups for whom the influenza vaccination is offered free of charge, while in other cases, it relates to groups for whom vaccination is recommended, but not offered for free. These distinctions are made based on the respective national health policies and guidelines available at the time of reporting. Additionally, the groups for which vaccination is recommended or offered do not always match the groups for which the country monitors coverage rates.

**Australia**

According to the Department of Health and Aged Care, annual influenza vaccination is recommended for everyone ≥6 months of age. In particular, it is recommended for:

- children aged 6 months to <5 years
- adults aged ≥65 years
- Aboriginal and Torres Strait Islander people
- people with medical conditions that increase their risk of severe influenza
- homeless people
- pregnant women
- healthcare workers, carers and household contacts of people in high-risk groups
- residents, staff, volunteers and visitors to aged care and long-term residential facilities
- commercial poultry and pork industry workers
- people who provide essential community services
- people who are travelling during influenza season

Source: <https://immunisationhandbook.health.gov.au/contents/vaccine-preventable-diseases/influenza-flu>. Accessed June 2024

**Canada**

The National Advisory Committee on Immunization (NACI) recommends that all individuals aged 6 months and older get the annual seasonal influenza vaccine, especially for populations at increased risk for influenza-related complications or hospitalization incluing:

- Pregnant people
- Children 6 months to less than 5 years of age
- Adults and children with certain chronic medial conditions (CMC), such as heart conditions, diabetes, cancer or immune disorder, anemia, renal diseases and morbid obesity
- Seniors 65 years and older
- Indigenous people

Source: <https://www.canada.ca/en/public-health/services/diseases/flu-influenza/get-your-flu-shot.html#a2>. Accessed June 2024.

**England**

Based on the UK Health Security Agency guidance, influenza vaccination is recommended to:

- everyone aged 65 years and over
- everyone under 65 years of age who has certain medical condition (e.g. heart, kidney or liver diseases, immunodeficiencies, diabetes), including children and babies over 6 months of age
- all pregnant women, at any stage of pregnancy
- all children aged 2 and 3 years (provided they were aged 2 or 3 years on 31 August before flu vaccinations starts in the autumn)
- all children in primary school
- some secondary school-aged children (Years 7 to 11)
- everyone living in a residential or nursing home
- everyone who receives a carer’s allowance, or are the main carer for an older or disabled person
- all those living with someone who has lowered immunity due to disease or treatment
- all frontline health and social care workers

Source: <https://www.gov.uk/government/publications/flu-vaccination-who-should-have-it-this-winter-and-why/the-flu-vaccination-who-should-have-it-and-why-winter-2023-to-2024>. Accessed June 2024.

**France**

Influenza vaccination is recommended for:

- people aged 65 years and over
- people under 65 years of age, including children from six months of age, suffering from certain chronic diseases;
- pregnant women;
- people suffering from obesity (BMI 40 or more)
- people staying in a residential care facility irrespective of their age
- healthcare professionals and any professional in regular and prolonged contact with people at risk of severe influenza
- people living in the same people with children under 6 months of age at risk of a serious complication of influenza, or immunocompromised people

Source: <https://www.service-public.fr/particuliers/actualites/A15259?lang=en>. Accessed June 2024.

**Germany**

The Standing Committee on Regular Vaccination (STIKO) recommends vaccination against flu for people at increased risk of severe illnesses or complications from the flu virus. This includes:

- children, teenagers and adults with underlying illnesses, such as chronic respiratory disorders, heart or cardiovascular disorders, metabolic disorders, congenital or acquired immune deficiencies,
- people aged 60 or older
- residents of old people’s homes or care homes
- pregnant women
- personnel in organisations with frequent contact with the public
- healthcare professionals
- people who may present a potential source of infection for people at risk living in or cared for by them in the same household.

Source: <https://www.impfen-info.de/grippeimpfung>. Accessed June 2024.

**Israel**

The vaccination is recommended for the entire population from the age of 6 months and older. In particular, it is highly recommended for:

- patients with long-term diseases (e.g. acquired or congenital heart disease, chronic lung diseases, diabetes, other chronic metabolic diseases, neurological or haematological diseases, diseases accompanied by immunosuppression, including AIDS or malignancies, patients receiving treatment that suppresses the immune system, …)
- women who intend to become pregnant, pregnant women, postnatal women
- children aged 6 months up to 18 years old who receive long-term aspirin therapy
- 50-year-olds and older, especially 65-year-old and above
- people living in non-open or semi-open institutions, with increased risk of infection
- healthcare workers

Source: <https://www.gov.il/en/pages/disease-flu?chapterIndex=2>. Accessed June 2024.

**Italy**

Based on the recommendations offered by the Italian Ministry of Health, the categories for which seasonal influenza vaccination is recommended are:

- People at higher risk of influenza-related complication or hospitalizations
  - People aged 65 years and older
  - women in any semester of pregnancy or in postpartum period
  - People aged 6 months to 65 years with diseases that increase the risk of complications from influenza
  - children and adolescents on long-term treatment with acetylsalicylic acid at risk of Reye’s syndrome
  - individual of any age living in long-term care facilities
  - Family members and contacts (adults and children) of people at high risk for complications (regardless of whether the person at risk has or has not been vaccinated)
- Individuals employed in public services of primary public interest and categories of workers (healhcare workers, police, firefighters, other socially useful categories)
- Personnel who, because of their work, are in contact with animals that could constitute a source of infection with non-human influenza viruses
- Other categories
  - Blodd donors
  - Healthy children in the age group 6 months to 6 years
  - People in the age group 60-64 years

Source: <https://www.aifa.gov.it/en/-/aifa-vaccini-influenzali-stagione-2023-2024>. Accessed June 2024.

**Japan**

According to the Ministry of Health, Labour and Welfare, people eligible for routine vaccination for influenza are:

- individuals aged 65 or over
- individuals aged 60-64 with dysfunction of the heart, kidney, or respiratory organs and are restricted in daily activities
- individual aged 60-64 with immune system disorders due to HIV infection and are not able to carry out normal daily activities

Source: <https://www.mhlw.go.jp/bunya/kenkou/kekkaku-kansenshou01/qa_eng.html>. Accessed June 2024.

**Netherlands**

The target group who can get the free flu vaccine includes:

- People aged 60 or older
- Children and adults with certain health problems (e.g. chronic heart disorder, diabetes mellitus, chronic kidney disease, reduced resistance to infection (e.g., because of (functional) asplenia, auto-immune disease, liver cirrhosis, chemotherapy or immunosuppressive medication), obesity (a BMI of 40 or higher)
- Children aged between 6 months and 18 years who are long-term salicylate users
- Women who are 22 weeks or more pregnant
- People with an intellectual disability
- People who are living in a nursing home, who are not included in one of the above categories

Source: <https://www.rivm.nl/en/flu-and-flu-vaccine/vaccine>. Accessed June 2024.

**South Korea**

Not available/Not found

**Spain**

The following population groups are recommended to be vaccinated against influenza:

- People at increased risk of complications in case of infections:
  - People 60 years of age and older
  - People 5 years or older in healthcare settings or long-term care facilities
  - People under 60 years of age with certain chronic conditions
  - Pregnant women in any trimester of pregnancy and women up to 6 months after delivery
  - People living with immunodepressed persons
- People working in critical or essential services for the community
  - healthcare workers
  - people working in essential public services, eg state security forces, firefighters, civil protection services
- Children between 6-59 months of age
- People 5 to 59 years of age at increased risk of complications from influenza (long-term therapy with acetylsalicylic acid, smokers)
- People with direct occupation exposure to animals (farmers, veterinarians, etc.)

[**https://www.sanidad.gob.es/areas/promocionPrevencion/vacunaciones/gripe_covid19/docs/RecomendacionesVacunacion_Gripe-Covid19.pdf**](https://www.sanidad.gob.es/areas/promocionPrevencion/vacunaciones/gripe_covid19/docs/RecomendacionesVacunacion_Gripe-Covid19.pdf)**.** Accessed June 2024.

**USA**

Routine annual influenza vaccination is recommended for all persons aged ≥6 months who do not have contraindications. If supply is limited, see priority groups in the ACIP statement.

Source: [**https://www.cdc.gov/flu/professionals/acip/summary/summary-recommendations.htm**](https://www.cdc.gov/flu/professionals/acip/summary/summary-recommendations.htm)**.** Accessed June 2024.
ACIP statement: <https://www.cdc.gov/mmwr/volumes/72/rr/rr7202a1.htm?s_cid=rr7202a1_w>. Accessed June 2024.
